# STAT1 and STAT3 Exhibit a Crosstalk and Are Associated with Increased Inflammation in Hepatocellular Carcinoma

**DOI:** 10.3390/cancers14051154

**Published:** 2022-02-23

**Authors:** Carolin Ploeger, Johannes Schreck, Thorben Huth, Angelika Fraas, Thomas Albrecht, Alphonse Charbel, Junfang Ji, Stephan Singer, Kai Breuhahn, Stefan Pusch, Bruno Christian Köhler, Christoph Springfeld, Peter Schirmacher, Arianeb Mehrabi, Benjamin Goeppert, Stephanie Roessler

**Affiliations:** 1 Institute of Pathology, Heidelberg University Hospital, 69120 Heidelberg, Germany; carolin.ploeger@med.uni-heidelberg.de (C.P.); johannes.schreck@med.uni-heidelberg.de (J.S.); thorben.huth@med.uni-heidelberg.de (T.H.); angelika.fraas@med.uni-heidelberg.de (A.F.); thomas.albrecht@med.uni-heidelberg.de (T.A.); alphonse.charbel@med.uni-heidelberg.de (A.C.); kai.breuhahn@med.uni-heidelberg.de (K.B.); peter.schirmacher@med.uni-heidelberg.de (P.S.); benjamin.goeppert@med.uni-heidelberg.de (B.G.); 2 Liver Cancer Center Heidelberg (LCCH), 69120 Heidelberg, Germany; bruno.koehler@nct-heidelberg.de (B.C.K.); christoph.springfeld@med.uni-heidelberg.de (C.S.); arianeb.mehrabi@med.uni-heidelberg.de (A.M.); 3 The MOE Key Laboratory of Biosystems Homeostasis & Protection, Zhejiang Provincial Key Laboratory for Cancer Molecular Cell Biology, Life Sciences Institute, Zhejiang University, Hangzhou 310058, China; junfangji@zju.edu.cn; 4 Institute of Pathology and Neuropathology, Eberhard-Karls University, 72076 Tuebingen, Germany; stephan.singer@med.uni-tuebingen.de; 5 Department of Neuropathology, Institute of Pathology, University Hospital Heidelberg, 69120 Heidelberg, Germany; s.pusch@dkfz-heidelberg.de; 6 Clinical Cooperation Unit Neuropathology, German Cancer Research Center (DKFZ), 69120 Heidelberg, Germany; 7 Department of Medical Oncology, National Center for Tumor Diseases, Heidelberg University Hospital, 69120 Heidelberg, Germany; 8 Department of General, Visceral and Transplantation Surgery, Heidelberg University Hospital, 69120 Heidelberg, Germany

**Keywords:** STAT1, STAT3, interleukin-6, liver cancer, tumor microenvironment

## Abstract

**Simple Summary:**

Liver cancer is the fourth-leading cause of cancer-related mortality worldwide and lacks effective therapies. Hepatocellular carcinoma (HCC) and cholangiocarcinoma (CCA) are the two most common types of liver cancer and both are associated with underlying inflammatory diseases. Thereby, interleukin-6 (IL-6)-mediated STAT3 signaling is critically involved in early carcinogenesis and disease progression. Here, we assessed the interplay between STAT1 and STAT3 in IL-6 signaling in vitro and studied the activation of STAT1 and STAT3 in a cohort of 124 HCC and a cohort of 138 CCA patients by immunohistochemistry. We found that IL-6 induced STAT1 transcriptional activity upon STAT3 depletion, suggesting that HCC tumor cells may activate both STAT1 and STAT3 signaling under pro-inflammatory conditions. Furthermore, HCC patient tissues showed a strong positive correlation of STAT1 and STAT3 activation in distinct patient groups. These patients also exhibited a high degree of immune cell infiltration, suggesting that these tumors are immune “hot”.

**Abstract:**

Liver cancers, which are mostly hepatocellular carcinoma (HCC) and cholangiocarcinoma (CCA), are very aggressive tumors with poor prognosis. Therapeutic options with curative intent are largely limited to surgery and available systemic therapies show limited benefit. Signal transducer and activator of transcription 1 (STAT1) and 3 (STAT3) are key transcription factors activated by pro-inflammatory cytokines such as interferon-γ (IFN-γ) and interleukin-6 (IL-6). In this study, we combined in vitro cell culture experiments and immunohistochemical analyses of human HCC (*N* = 124) and CCA (*N* = 138) specimens. We observed that in the absence of STAT3, IL-6 induced the activation of STAT1 and its target genes suggesting that IL-6 derived from the tumor microenvironment may activate both STAT1 and STAT3 target genes in HCC tumor cells. In addition, STAT1 and STAT3 were highly activated in a subset of HCC, which exhibited a high degree of infiltrating CD8- and FOXP3-positive immune cells and PD-L1 expression. Our results demonstrate that STAT1 and STAT3 are expressed and activated in HCC and tumor infiltrating immune cells. In addition, HCC cases with high STAT1 and STAT3 expression also exhibited a high degree of immune cell infiltration, suggesting increased immunological tolerance.

## 1. Introduction

Despite growing efforts over the last two decades, liver cancer is the fourth-leading cause of cancer-related mortality worldwide [1,2]. Hepatocellular carcinoma (HCC) is the most common type of liver cancer, followed by cholangiocarcinoma (CCA), which accounts for 10–15% of liver cancers [2,3]. Both HCC and CCA can be caused by chronic hepatobiliary diseases such as chronic infection with hepatitis B (HBV) and C viruses (HCV) or other inflammatory liver diseases such as alcoholic and non-alcoholic steatohepatitis and primary sclerosing cholangitis [4], thus making HCC and CCA a paradigm for inflammation-induced carcinogenesis [5,6,7]. Thereby, tumor cells are growing in a complex microenvironment of tumorous and non-tumorous cells, and secreted small molecules [5,6]. However, the interplay between tumor cells and the tumor microenvironment including infiltrating immune cells is still poorly understood.

Tumor cells are highly effective in escaping from immune-mediated eradication and they may even induce tumor promoting factors in the tumor microenvironment [8]. This interplay of tumor cells with components of their microenvironment may induce pro-tumorigenic pathways, enhancing tumor progression and leading to poor outcome [9]. In HCC and other cancer types, several studies have shown the critical involvement of inflammation, especially of interleukin-6 (IL-6) signaling during carcinogenesis [10,11,12]. In an HCC mouse model of diethylnitrosamine (DEN)-induced tumorigenesis, ablation of IL-6 expression led to lower HCC incidence in male mice, whereas female mice did not show this effect due to estrogen-mediated inhibition of IL-6 secretion [13]. Supporting these data, high serum IL-6 levels were found to be associated with rapid progression from chronic viral hepatitis to HCC in HBV- and HCV-positive patients [14,15]. Therefore, it is crucial to decipher the role of IL-6 signaling in the interplay between tumor cells and the tumor-microenvironment for the development of novel treatment modalities.

Signal transducer and activator of transcription 3 (STAT3) is the main transcription factor mediating IL-6-induced signaling in tumor and immune cells, whereas interferon-γ (IFN-γ) signaling activates the closely related STAT1 protein [16]. A crosstalk between STAT1 and STAT3 has been proposed with STAT1 and STAT3 playing opposite roles in tumorigenesis [17,18]. They both modulate tumor angiogenesis, invasion, and anti-tumor immune response in an opposing manner. STAT1 is considered to be a tumor suppressor while STAT3 is rather seen as an oncogene [19,20,21]. Nevertheless, hepatoprotective functions of STAT3 during liver damage have also been reported [22]. In the tumor and its microenvironment, STAT1 and STAT3 have both been shown to be expressed by tumor cells and infiltrating immune cells and to be involved in regulating cancer adaptive immunity [21]. Recently, we demonstrated that immune cell infiltration is associated with downregulation of the tumor suppressor SH2 Domain Containing 4A (SH2D4A), which was able to suppress IL-6/STAT3 signaling [23,24]. However, the interplay between STAT1 and STAT3 signaling in liver cancer cells and the immune cell composition regarding STAT1 and STAT3 expression in the tumor cells is still unclear. Therefore, we analyzed the loss of STAT1 and STAT3 in HCC cell lines and the resulting downstream signaling effect. Furthermore, in this study, we aimed at dissecting the activation of STAT1 and STAT3 in tumor cells and the immune cell infiltrate in human HCC tissue samples.

## 2. Materials and Methods

### 2.1. Tissue Microarrays

Tissue microarrays (TMA) including 124 HCC and 138 CCA tumor tissue samples were used. HCC tumor tissues of 124 patients who were surgically resected between 2006 and 2011 at the University Hospital of Heidelberg and histologically classified according to established criteria by two experienced pathologists independently (Table S1). Furthermore, CCA tissue samples were obtained from patients undergoing surgery at Heidelberg University Hospital between 1995 and 2010. In total, the CCA cohort consisted of 138 patients: 61 with intrahepatic CCA (iCCA), 45 with perihilar CCA (pCCA), and 32 with distal CCA (dCCA; Table S2). Both cohorts have been used previously [24,25,26,27,28]. Inclusion of tumor tissue for this study was approved by the institutional ethics committee (S-206/2005 and S-519/2019). The study was supported by the tissue bank of the National Center for Tumor Diseases (NCT, Heidelberg, Germany).

### 2.2. Tissue Microarray Construction

For generation of tissue microarrays, 3 μm sections were cut and stained with hematoxylin and eosin (H&E). Representative areas from the tumor center and non-neoplastic bile duct tissue of the respective region were marked by two experienced pathologists. Tumor tissue cores with 0.6 mm diameter for the HCC or with 1.5 mm diameter for the CCA TMAs were consecutively punched out of the sample tissue blocks and embedded into a new paraffin array block using a tissue microarrayer (Beecher Instruments, Woodland, CA, USA).

### 2.3. Immunohistochemistry

For the detection of STAT1, a monoclonal mouse IgG antibody directed against STAT1 (clone number C-136; dilution 1:200; Santa Cruz Biotechnology, Dallas, TX, USA) and for the detection of STAT3, a monoclonal rabbit IgG antibody directed against STAT3 (clone number 79D7; dilution 1:400; Cell Signaling Technology, Danvers, MA, USA), were used. Staining was performed on an automated system (Discovery Ultra, Ventana, Tuscon, AZ, USA) following the manufacturer’s instructions using Dako Target Retrieval Solution, Citrate pH 6 (S2369, Agilent Technologies, Santa Clara, CA, USA) and the Dako Target Retrieval Solution, pH 9 (S2367, Agilent Technologies), respectively. For staining of PD-L1, anti-PD-L1 (ready-to-use, clone SP263, Roche Diagnostics, Rotkreuz, Switzerland) antibody was used. Three µm sections of the TMA were deparaffinized, pre-treated with an antigen retrieval buffer (Tris/Borat/EDTA, pH 8.4; Ventana, Roche), and stained using an automated device (Ventana Benchmark Ultra, Roche). Immunohistochemistry to detect and count specific inflammatory cells using antibodies specific to CD3, CD4, CD8, CD20, CD68, CD117, and FOXP3 was performed as recently described [24,25].

### 2.4. Tissue Microarray Evaluation

STAT1 and STAT3 expression were scored by applying a semi-quantitative immunoreactive score (IRS), resulting in the expression of values ranging from 0 (no expression) to 12 (strong expression in more than 80% of tumor cells), as previously described [29]. Briefly, staining intensity (0: no staining, 1: weak staining, 2: moderate staining, 3: strong staining) as well as percentage of positively stained cells (0: no cells stained, 1: up to 10% of cells stained, 2: 10–50% of cells stained, 3: 51–80% of cells stained, 4: more than 80% of cells stained) were scored separately and the IRS for each individual case was calculated by multiplication of the intensity and the percentage score. PD-L1 expression was categorized by tumor proportion score (TPS), which was defined as the percentage of tumor cells with membranous PD-L1 staining based on all tumor cells, and combined positive score (CPS), defined as the number of PD-L1-positive cells (tumor cells, lymphocytes, and macrophages) divided by total number of tumor cells × 100%. Patients were assigned to the high expression group with PD-L1 TPS or CPS larger or equal 1%. Immunohistochemical staining of immune cell markers was performed and analyzed previously, as described. [24,25,30]. These data on immune cell infiltration were thus derived from previous studies. For technical reasons, some tissue microarray dots could not be evaluated due to loss of tissue or staining artifacts. Therefore, the number of included cases for the immunohistochemical analysis of STAT1 and STAT3 expression and for the immune cell types varied slightly. 

### 2.5. Cell Lines and siRNA-Mediated Knockdown

Cell lines were obtained from ATCC (HepG2) or JCRB (HuH1 and HuH7), regularly tested for mycoplasma contamination (MycoAlert, Lonza, Basel, Switzerland), and authenticated by STR analysis. HuH1 and HuH7 cells were cultured in Dulbecco’s modified Eagle’s medium (DMEM) and HepG2 in RPMI-1640 medium. All growth media were supplemented with 10% FCS and 1% penicillin/streptomycin (Thermo Fisher Scientific, Waltham, MA, USA). For siRNA-mediated knockdown of STAT1 or STAT3, Lipofectamine RNAiMAX Transfection Reagent (Thermo Fisher Scientific) was used according to the manufacturer’s instructions. siRNAs were obtained from Qiagen and are listed in Supplementary Table S3.

### 2.6. RNA Extraction, cDNA Synthesis and Semi-Quantitative Reverse-Transcription Polymerase Chain Reaction (RT-PCR)

Total RNA was extracted from liver cancer cell lines by applying the NucleoSpin RNA Kit (Macherey-Nagel, Düren, Germany) according to the manufacturer’s protocols. cDNA was synthesized from 500 ng total RNA using the RevertAid H Minus First Strand cDNA Synthesis Kit (Thermo Fisher Scientific). Samples were analyzed in triplicate using primaQUANT CYBR-Green Mastermix low ROX (Steinbrenner Laborsysteme, Wiesenbach, Germany) on a StepOnePlus real-time PCR instrument (Applied Biosystems, Darmstadt, Germany). The reference gene *Serine and Arginine rich Splicing Factor 4* (*SRSF4*) was used as the normalization control. Relative mRNA expression values were calculated using the comparative Ct method. Primers were obtained from Thermo Fisher Scientific and Apara-Bioscience (Denzlingen, Germany) and are listed in Supplementary Table S4.

### 2.7. Protein Isolation and Western Blot

Total protein was extracted from cells using cell lysis buffer 10× (Cell Signaling Technology) supplemented with phosphatase inhibitor PhosStop and protease inhibitor Complete Mini EDTA-free (both Roche Diagnostics, Mannheim, Germany). Protein concentrations were determined using the Bradford assay (Sigma Aldrich, Taufkirchen, Germany). Protein samples were prepared in equal amounts with water and 4× loading buffer (250 mM Tris pH 6.8, 8% SDS, 40% glycerol, 100 mM DTT, 0.04% bromophenol blue). Twenty µg of protein was separated on 8% to 12% Bis/Tris-polyacrylamide gels and then transferred to an equilibrated nitrocellulose membrane (Merck Chemicals, Darmstadt, Germany). Membranes were blocked with 5% milk in Tris-buffered saline with Tween 20 (TBST) or 5% bovine serum albumin (BSA) in TBST and incubated with the indicated primary antibodies (Supplementary Table S5) overnight at 4 °C. Proteins were detected with IRDye secondary antibodies using an Odyssey Sa Infrared Imaging System (LI-COR Biosciences, Bad Homburg, Germany). Protein bands were quantified by densitometry using the Image Studio Software v.3.1.4 (LI-COR) and normalized to loading control β-Actin or β-Tubulin, as indicated. Whole Western blot images are provided in the Supplemental online.

### 2.8. Luciferase Reporter Assay

To analyze the impact of IL-6 on STAT1 transcriptional activity in STAT3-depleted cells, luciferase reporter assays were performed. HepG2 and HuH7 cells were seeded in 24-well plates and transfected with respected siRNAs by using Lipofectamine RNAiMAX Transfection Reagent. Twenty-four hours after siRNA transfection, cells were co-transfected with Firefly luciferase reporter vector pGL4 [luc2P/GAS-RE/Hygro] and pRL-TK (Renilla luciferase control reporter vector). The next day, cells were treated for 24 h with 20 ng/mL IL-6 and luciferase activity was analyzed by the Dual-Luciferase Reporter Assay System (Promega, Mannheim, Germany) according to the manufacturer’s protocol using an Omega FLUOstar Microplate Reader (BMG LABTECH GmbH, Ortenberg, Germany). Renilla luciferase was used as the internal transfection control and for normalization.

### 2.9. Statistical Analysis

In the case of two group comparisons, differences were assessed using the nonparametric Mann–Whitney U test. The association of two variables was assessed using nonparametric Spearman’s correlation analysis. Statistical analyses were performed with GraphPad Prism 6.0 (GraphPad Software, La Jolla, CA, USA) and the statistical computing environment R version 4.0.3 (http://www.R-project.org/, released 10 October 2020, last accessed 10 January 2022). For data analysis, R package Hmisc was used and plots were generated by package Corrplot. *p*-values below 0.05 were considered statistically significant.

## 3. Results

### 3.1. STAT1 Depletion Does Not Induce an IL-6-Like Response in HCC Cells

**Figure 1:**
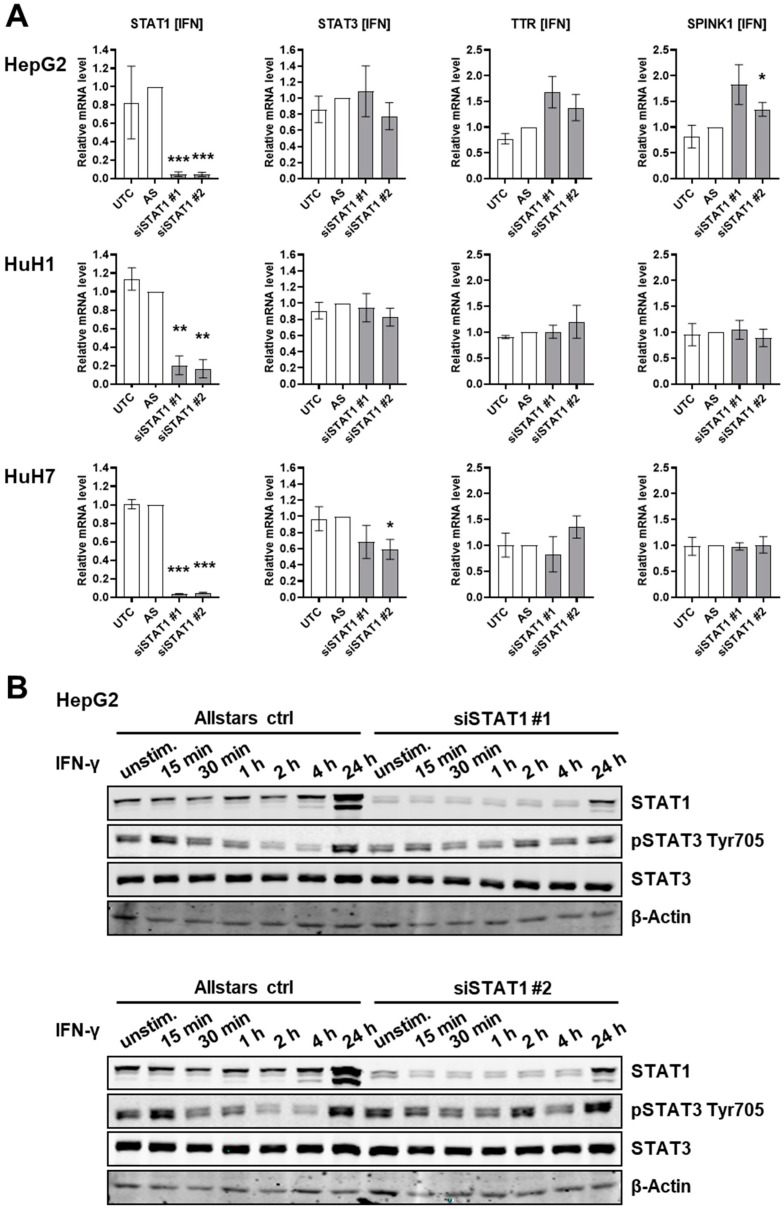
STAT1 depletion does not induce an IL-6-like response in HCC cells. (**A**) STAT1 was depleted using two different siRNAs, siSTAT1#1 and siSTAT1#2, in HepG2, HuH1, and HuH7 cells (as indicated) and cells were incubated with IFN-γ (500 U/mL, 24 h). Relative RNA expression values are shown for *STAT1*, *STAT3*, *TTR*, and *SPINK1*. Untreated control (UTC), Allstars control (AS), *n* = 3, * *p* < 0.05, ** *p* < 0.01, *** *p* < 0.001. (**B**) Western blot detecting STAT1, STAT3, and pSTAT3 Tyr705 in the control or STAT1-depleted HepG2 cells upon incubation with IFN-γ (500 U/mL) for indicated time points. β-Actin served as the loading control.

To assess the interplay of STAT1 and STAT3 protein in liver cancer cells, we first investigated the effect of STAT1 protein knockdown in the three liver cancer cell lines HepG2, HuH1, and HuH7, which all express the STAT1 and STAT3 proteins (Figure 1 and Supplementary Figure S1). By using two independent siRNAs against STAT1, its mRNA expression was depleted >90% in the different liver cancer cell lines (Figure 1A). Expression of IL-6/STAT3 target genes Transthyretin (TTR) and Serine Peptidase Inhibitor Kazal Type 1 (SPINK1) showed that STAT1 depleted cells treated with IFN-γ, a known inducer of the STAT1-signaling pathway, exhibited no significant induction of JAK/STAT3-pathway activation at the transcriptional level in all three cell lines (Figure 1A). At protein level, STAT1-depleted HepG2 cells showed a slightly stronger Tyr705 phosphorylation of STAT3 (pSTAT3 Tyr705) after 2 h of IFN-γ stimulation compared to Allstars transfection control (Figure 1B, top and bottom panel). However, the activation of STAT3 by Tyr705 phosphorylation did not persist and was not observed in HuH1 (Figure S1A) and HuH7 (Figure S1B) cells, suggesting that STAT1 depletion does not induce an IL-6-like response in HCC cells. Therefore, we did not observe any significant effect of STAT1 protein depletion on STAT3 protein activation or on STAT3 target gene expression in three different liver cancer cell lines.

### 3.2. STAT3 Depletion Induces an IFN-γ-Like Response in HCC Cells

**Figure 2:**
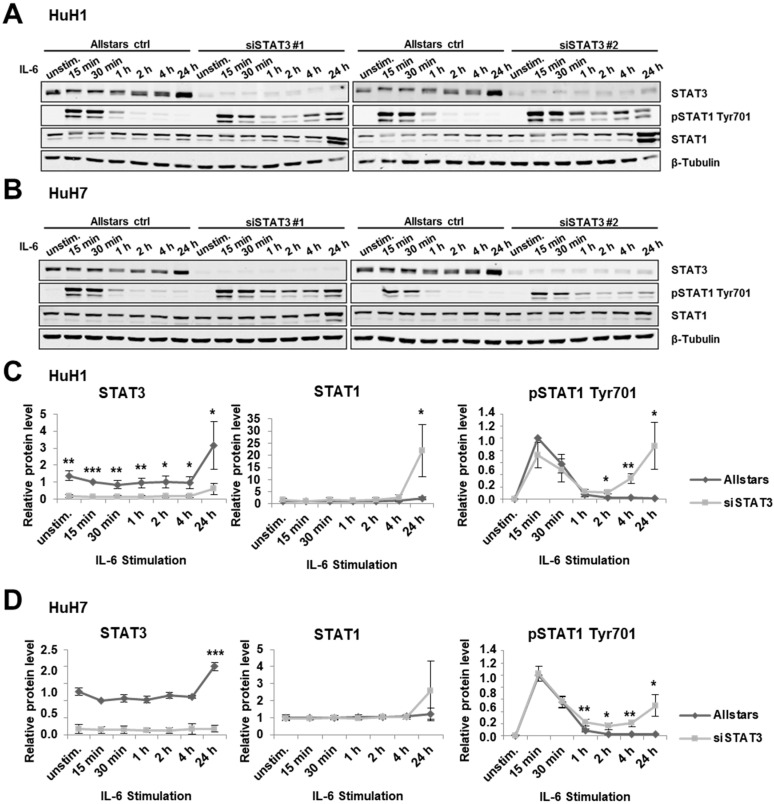
STAT3 depletion prolongs IL-6-induced STAT1 phosphorylation. (**A**,**B**) Western blot of STAT3, pSTAT1 Tyr701, and STAT1 proteins are shown. STAT3 was depleted using two different siRNAs, siSTAT3 #1 and siSTAT3 #2 in (**A**) HuH1 and (**B**) HuH7 cells. Cells were incubated with IL-6 (20 ng/mL) for indicated time points. β-Tubulin served as loading control. (**C**,**D**) Relative quantification of STAT3, STAT1, and pSTAT1 Tyr701 protein expression levels at indicated time points from two independent experiments in (**C**) HuH1 and (**D**) HuH7 cells. Results of siSTAT3 #1 and siSTAT3 #2 are combined. * *p* < 0.05, ** *p* < 0.01, *** *p* < 0.001.

Next, we performed the opposite experiment by depleting STAT3 protein expression followed by IL-6 treatment. In three different liver cancer cell lines, HepG2, HuH1, and HuH7, siRNA-mediated STAT3 depletion prolonged STAT1 phosphorylation at Tyr701 upon IL-6 treatment compared to Allstars transfection control (Figure 2 for HuH1 and HuH7 and Supplementary Figure S2A for HepG2). In control cells, short-term activation of STAT1 represented by phosphorylated STAT1 at Tyr701 decreased from 1 h after stimulation and stayed very low. In contrast, pSTAT1 Tyr701 was still observed at 2 h, 4 h, and 24 h time points in STAT3-depleted cells. Interestingly, after 24 h, the expression of total STAT1 was increased compared to the control cells, indicating a positive feedback mechanism (Figure 2).

### 3.3. IL-6 Induces STAT1 Transcriptional Activity upon STAT3 Depletion

**Figure 3:**
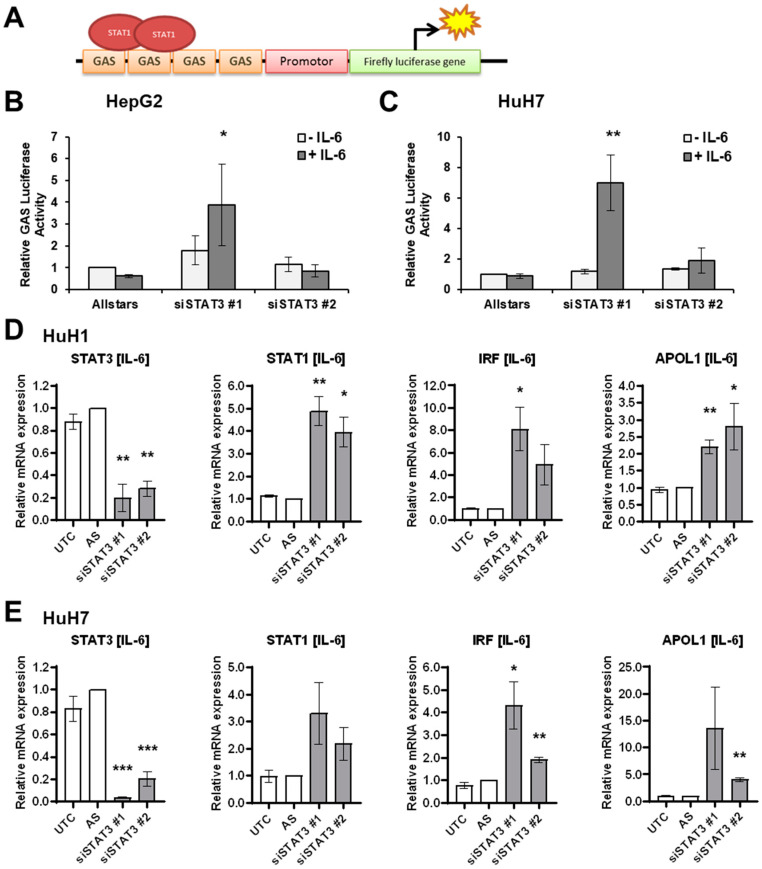
IL-6 induces STAT1 transcriptional activity upon STAT3 depletion. (**A**) To quantitatively measure STAT1 transcriptional activity, a luciferase reporter containing four interferon-gamma activated sites (GAS) was used. (**B**,**C**) Luciferase assay in (**B**) HepG2 and (**C**) HuH7 cells measuring endogenous STAT1 transcriptional activity using the GAS-element containing reporter co-transfected with Allstars (control), siSTAT3 #1, or siSTAT3 #2, in the presence or absence of 20 ng/mL IL-6 for 24 h, as indicated. Renilla luciferase was used as the internal transfection control and for normalization. Data were normalized to IL-6 unstimulated transfection control cells (mean ± SD). (**D**,**E**) Relative mRNA expression levels of *STAT3*, *STAT1*, and the STAT1 target genes *IRF1* and *APOL1* measured by semi-quantitative RT-PCR in (**D**) HuH1 and (**E**) HuH7 cells upon transfection with Allstars control siRNAs (AS), siSTAT3 #1, or STAT3 #2 in the presence of 20 ng/mL IL-6 for 24 h. *N* = 3. * *p* < 0.05, ** *p* < 0.01, *** *p* < 0.001.

Next, we aimed to analyze the impact of STAT3 depletion on STAT1 transcriptional activity by applying the luciferase assay (Figure 3A). While control cells showed no transcriptional activation upon IL-6 treatment, STAT3-depleted cells showed increased activity using siSTAT3#1 (Figure 3B,C). However, siSTAT3#2 did not show any increased luciferase activity potentially due to inefficient deletion of STAT3 protein (Figure 2A,B and Figure 3D,E). To further evaluate the effect of potential STAT1 transcriptional activity upon STAT3 depletion, we evaluated the gene expression levels of well-known STAT1 target genes *Interferon Regulatory Factor 1* (*IRF1*) and *Apolipoprotein L1* (*APOL1*). In HuH1 and HuH7 cells, both STAT1 target genes were significantly upregulated upon IL-6 stimulation in STAT3-depleted cells (Figure 3D,E for HuH1 and HuH7; Supplementary Figure S2B for HepG2). Interestingly, *STAT1* expression was also significantly induced, suggesting a positive feedback loop (Figure 3D,E). Therefore, downregulation of STAT3 protein levels results in increased transcriptional activity of STAT1 in the presence of IL-6 and prolonged activation of STAT1 signaling.

### 3.4. Expression of STAT1 and STAT3 Strongly Correlates with Infiltrating Immune Cell Profiles

Immune cell infiltration has been reported to influence epithelial tumor cells [21]. As we found that IL-6 induces STAT1 transcriptional activity upon STAT3 depletion, we next sought to analyze the expression of STAT1 and STAT3 regarding infiltrating immune cells in human liver cancer tissues. HCC (*N* = 124) and CCA (*N* = 138) tumor tissue microarrays were stained for STAT1 and STAT3 using immunohistochemistry (Tables S1 and S2). Comparison of STAT1 and STAT3 nuclear expression levels, respectively, in tumor cells revealed no significant differences in age, sex, etiology, liver disease, nodularity, vascular invasion, and grading in patients with HCC (Table S1). High nuclear expression of STAT3 protein was associated with male gender (*p* = 0.0002) and perihilar or distal localization (*p* = 0.001) in CCA but not with histology, grading, and tumor staging (Table S2). In addition, clinical characteristics of patients with CCA did not differ between STAT1 low and STAT1 high groups (Table S2).

**Figure 4:**
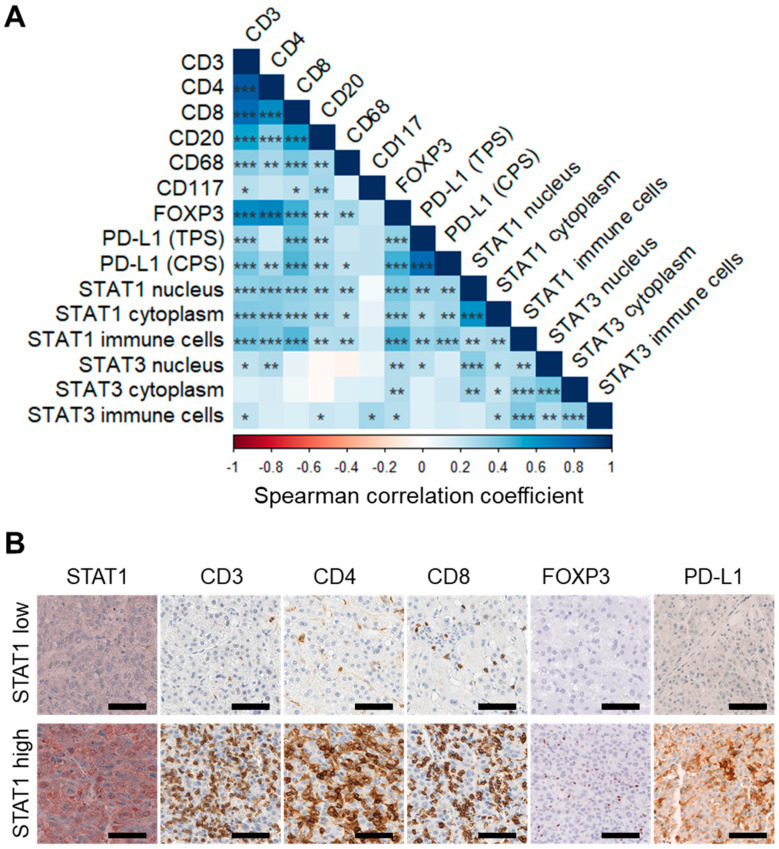
Correlation of STAT1 and STAT3 expression with immune cell infiltration in HCC. (**A**) Correlation matrix of immune cell infiltrates and immunohistochemical STAT1 or STAT3 expression in HCC tumor tissue samples. Red color indicates negative correlation, whereas blue color indicates positive correlation. Darker coloration indicates a higher Spearman correlation coefficient and asterisk denotes the level of significance. Spearman *p*-value: * <0.05; ** <0.01; *** <0.001. (**B**) Exemplary HCC tumor tissues with low or high immunohistochemical reaction against STAT1 are shown, respectively. The corresponding immune cell infiltrate stained for CD3, CD4, CD8, FOXP3, and PD-L1 protein expression in the same tumor dot is shown as indicated. Scale bar: 100 µm.

Next, T-cell populations were quantified by anti-CD3, anti-CD4, anti-CD8, and anti-FOXP3 staining. B cells, macrophages, and mast cells were detected using anti-CD20, anti-CD68, and anti-CD117 staining, respectively. To evaluate the potential association of STAT1 and STAT3 with specific infiltrating immune cell populations, we performed paired correlation analyses. Overall, strong positive correlations were observed in HCC (Figure 4), but fewer correlations were seen in CCA (Supplementary Figure S4). Thereby, total immune cell counts and intraepithelial immune cell counts exhibited similar effects in CCA (Supplementary Figure S4). In HCC, nuclear and cytoplasmic STAT1 expression, together with the STAT1-positive immune cell infiltrate, correlated highly significant with CD3-, CD4-, CD8-, and FOXP3-positive immune cell infiltrates (Spearman r = 0.335, 0.349, 0.348, and 0.394, all with *p* < 0.001; Figure 4A,B and Supplementary Figure S3). In addition, weaker, but significant correlations of nuclear and cytoplasmic STAT1 in the tumor cells and of STAT1-positive immune cells were observed with the CD20- and CD68-positive immune cell infiltrate (Figure 4A). Only few CD117-positive immune cells were detected and no significant correlation was observed for CD117-positive immune cell infiltrate with nuclear or cytoplasmic STAT1 expression of the tumor epithelium. In contrast, correlation coefficients for STAT3 were lower overall and reached significance in fewer pairings compared to STAT1. Thus, the highest correlation existed between epithelial nuclear STAT3 staining and CD4- or FOXP3-positive immune cells (Figure 4A).

**Figure 5:**
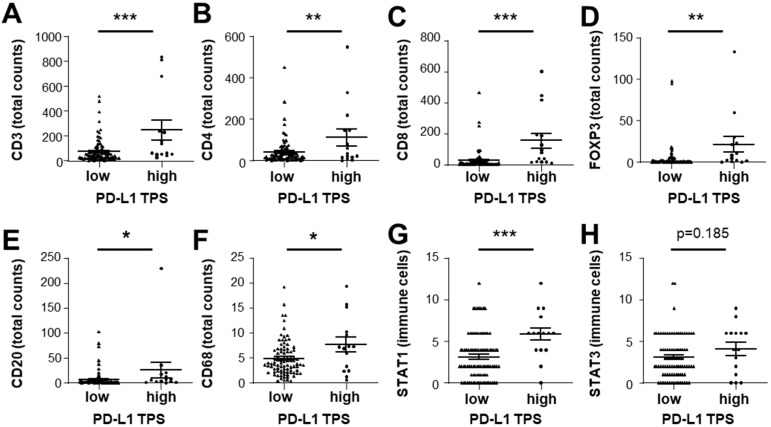
Association between PD-L1 expression in the tumor epithelium and immune cell infiltration in HCC tumor samples. (**A**) CD3-, (**B**) CD4-, (**C**) CD8-, (**D**) FOXP3-, (**E**) CD20-, (**F**) CD68-, (**G**) STAT1-, and (**H**) STAT3-positive immune cell infiltrate in HCC tumor tissue samples stratified by low (*N* = 90) and high PD-L1 TPS expression score (*N* = 15). TPS—tumor proportion score. * *p* < 0.05; ** *p* < 0.01; *** *p* < 0.001.

As tumors with high immune cell infiltration, also termed immune “hot” tumors, have been suggested to express the immune checkpoint molecule PD-L1 [31], we correlated the expression of PD-L1 with immune cell population markers and STAT protein expression (Figure 4). PD-L1 TPS, indicating tumor-specific, and PD-L1 CPS, indicating immune cell and tumor-specific staining, both strongly correlated with STAT1 expression and with cytotoxic CD8- and FOXP3-positive cells (Figure 4). Thus, PD-L1-positive HCC exhibited immune “hot” characteristics with significantly higher overall immune cell infiltration (Figure 5). Furthermore, when comparing HCC tissue samples by median of nuclear STAT3 expression, CD3-, CD4-, CD8-, and FOXP3-positive immune cell counts were significantly higher in tumor tissues with high nuclear STAT3 expression compared to tumor tissues with low nuclear STAT3 expression of the tumor cells (Figure S5). No difference between HCC tumor cells with high or low nuclear STAT3 tumor epithelium was detected regarding CD20-, CD68-, and CD117-positive immune cell infiltrates (Figure S5). Therefore, active STAT3 signaling in the tumor cells, evident by nuclear localization of STAT3, was associated with infiltrating FOXP3-T-cell numbers, suggesting that nuclear STAT3 may be associated with increased active immune response.

**Figure 6:**
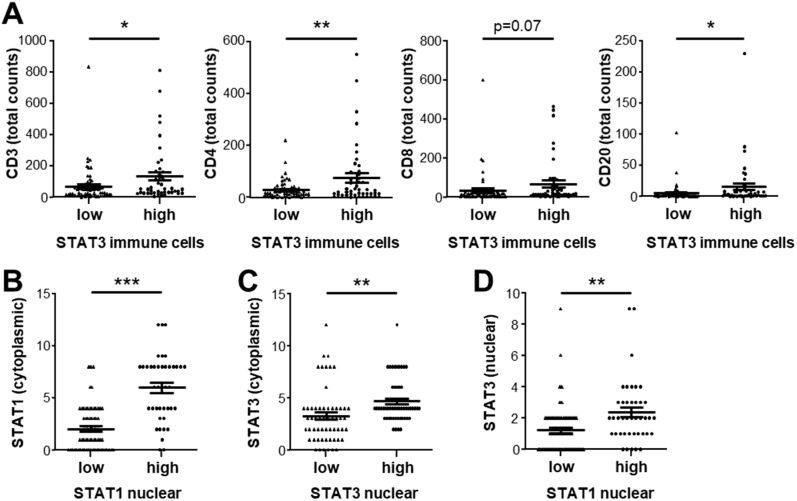
Association between STAT1 and STAT3 expression in the tumor epithelium and immune cell compartment in HCC tumor samples. (**A**) CD3-, CD4-, CD8-, and CD20-positive immune cell infiltrate in HCC tumor tissue samples stratified by median count of STAT3-positive immune cells. (**B**) Cytoplasmic STAT1 expression IRS in HCC tumor samples stratified by median nuclear STAT1 expression. (**C**) Cytoplasmic STAT3 expression IRS stratified by median nuclear STAT3 expression. (**D**) Nuclear STAT3 expression IRS stratified in patient samples grouped by median nuclear STAT1 expression. * *p* < 0.05; ** *p* < 0.01; *** *p* < 0.001.

To better understand the role of STAT3 in the tumor microenvironment, we compared HCC samples that were positive or negative for STAT3-expressing immune cells. Patient samples with high numbers (median cutoff) of STAT3-expressing immune cells had significantly higher infiltration of CD3-, CD4-, and CD20-positive immune cells (Figure 6A). Interestingly, STAT1 and STAT3 exhibited highly positive correlations in the tumor epithelium. Nuclear STAT1 was strongly and significantly correlated with cytoplasmic STAT1, nuclear STAT3, and cytoplasmic STAT3, and vice versa. HCC tissues with positivity for tumor cell nuclear STAT1 had high cytoplasmic STAT1, suggesting our in vitro data showed that prolonged activation of STAT1 phosphorylation results in increased total STAT1 protein levels (Figure 6B). Similarly, tumor tissue samples with high positivity for tumor cell nuclear STAT3 exhibited high cytoplasmic STAT3 levels and tumors with positivity for nuclear STAT1 showed increased nuclear STAT3 (Figure 6C,D). Therefore, activated STAT1 and activated STAT3 signaling in the tumor cells was observed in a subsets of HCC patients and may indicate a specific HCC subgroup with high immune cell infiltration.

## 4. Discussion

The tumor microenvironment plays a crucial role in cancer progression, and targeting the immune system by cancer immunotherapy has become a promising therapeutic option recently approved for HCC. However, in HCC and several other tumor entities, cancer immunotherapy is effective only in a small subpopulation of patients. Therefore, it is important to better understand the composition of distinct immune cell populations and their interaction with the tumor cells within the tumor microenvironment. This study focused on the role of STAT1 and STAT3 signaling in liver cancer. Using human cell lines, we demonstrated that IL-6 induces STAT1 transcriptional activity upon STAT3 depletion, suggesting that HCC cells may activate both STAT1 and STAT3 signaling under pro-inflammatory conditions. Consistently with these in vitro observations, HCC subgroups showed a great degree of positive correlation of STAT1 and STAT3 activation. These STAT1-positive tumors also exhibited a high degree of immune cell infiltration, especially CD4-, CD8, and FOXP3-positive T cells, suggesting that an active engagement of the immune system with the tumor takes place and that these tumors are immune “hot”. Our observation that STAT1-signaling and STAT1 target gene expression may be activated by IL-6 proposed that STAT3 and STAT1 signaling are interconnected in HCC. This is also supported by the positive correlation of STAT1 and STAT3 expression in human HCC samples in tumor and immune cells, indicating high immunological tolerance in a subset of HCC patients, as evident by PD-L1 expression.

Interestingly, STAT3 knockout in mouse embryo fibroblasts exhibited an IFN-γ-like response including an extended STAT1 activation upon IL-6 treatment (Costa-Pereira et al., 2002). In contrast, upon STAT1 loss, IFN-γ-mediated STAT3 activation was much stronger and prolonged, leading to the expression of some STAT1 target genes that are typically transcribed in IFN-γ-treated wild-type cells [32]. Similar effects were also seen in STAT1-deficient bone-marrow-derived macrophages and T lymphocytes [33]. Thus, our data in liver cancer cell lines are in line with previous studies in fibroblasts and ma-crophages whereby a disbalance between STAT1 and STAT3 may lead to the activation of IFN-γ/STAT1 target genes in a proinflammatory IL-6-containing tumor microenvironment. To some extent, this may explain the pro- and anti-tumorigenic effects of STAT1 signaling in liver carcinogenesis [18]. Furthermore, we could show that STAT1 depletion did not induce an IL-6-like response in HCC cells treated with IFN-γ, indicating distinct regulatory mechanisms. Taken together, STAT1 and STAT3 signaling pathways are tightly interconnected and IFN-γ/STAT1 target genes may be activated in a proinflammatory IL-6-containing tumor microenvironment.

Furthermore, we analyzed the expression of STAT1 and STAT3 protein in human HCC and CCA tissue samples separately. STAT1 and STAT3 protein expression were strongly and positively correlated in the tumor cells and infiltrating immune cells. This suggests that the stimulatory cytokines in the tumor microenvironment may equally act on the tumor cells and the immune cells, and that various cell types are susceptible for inflammatory signals.

Tumor-infiltrating T cells are a marker of increased immunological tolerance in cancer. Tumors with the so-called T cell–inflamed phenotype consisting of infiltrating T cells appear to resist immune attack through the dominant inhibitory effects of immune system–suppressive pathways [34]. In contrast, it is believed that tumors that lack T-cell infiltration may resist immune attack through immune system exclusion or ignorance [34]. A recent large-scale profiling study aiming to identify immune subtypes across 33 cancer types suggested six immune subtypes with prognostic relevance [9]. Thereby, substantial infiltration of CD8- and CD4-positive immune cells was observed in four out of six subtypes, stressing the importance and wide involvement of CD4- and CD8-positive T cells. Furthermore, infiltrating T cells have prognostic and therapeutic relevance. Higher presence of tumor-infiltrating T lymphocytes is generally considered a favorable prognostic factor [35]. In CCA, patients with intraepithelial tumor-infiltrating CD4-, CD8-, and FOXP3-T lymphocytes showed a significantly longer overall survival [25]. Similarly, significantly fewer patients with a high density of CD8-positive T-cell infiltrates experienced recurrence of their HCC within three years compared with those exhibiting a low CD8-cell density [36]. Despite the favorable prognosis of patients with T-cell infiltration, long-term survival of patients with or without T-cell infiltration is dismal.

FOXP3-positive regulatory T-cells (Treg) are believed to mediate the suppression of anti-tumor immunity, which may lead to more aggressive disease [37,38]. It has been suggested that STAT3 directly binds to a STAT consensus site in the FOXP3 promoter to enhance FOXP3 expression of Treg cells increasing their inhibitory function [39,40]. Here, we demonstrated that STAT3 expression and activation, evident by nuclear localization, strongly correlated with the presence of FOXP3-positive Tregs. The development of immune checkpoint inhibitors that block negative regulators of T-cell immunity provide powerful therapeutic options. However, immunotherapies are only effective in a small subset of liver cancer patients and a better understanding of the tumor microenvironment is crucial to improve patient outcome. Interestingly, STAT3 may directly bind the *PD-1* promoter and activate PD-1 protein expression in T cells [41]. Therefore, the degree of STAT3-positive immune cells in HCC may be linked to the response to immune checkpoint blockade.

Our observation that STAT1 and STAT3 expression exhibit strong positive correlation in the tumor cells and the immune cell infiltrate suggest that both may similarly indicate the state of the tumor microenvironment. Existing evidence suggests that JAK-STAT signaling members may ultimately serve as diagnostic markers stratifying patients that may benefit from more targeted therapeutic approaches that modulate downstream targets, rather than upstream JAK-STAT pathway regulators [42]. Targeting oncogenic transcription factors of the STAT family has been suggested as powerful approach potentially modulating gene regulatory processes including chromatin remodeling [43]. In contrast to other tumor entities, PD-L1 blockage did not significantly prolong HCC patient survival, suggesting that PD-L1/PD-1 axis blockade alone may not be sufficient to initiate adequate levels of anticancer immunity in HCC [44,45]. Recently, it has been demonstrated that inhibition of STAT3 leads to a reduction in PD-L1 protein expression, which may constrain tumoral inflammation and improve immune response against tumor cells [46,47]. Furthermore, we were able to show that PD-L1 and STAT1 expression correlated in both the tumor cells and the tumor infiltrating immune cells. Interestingly, IL-6 knockdown in cancer associated fibroblasts (CAF) increased IFN-γ on CD8-postive T cells and IL-6 blockade could reverse anti-PD-L1 resistance in an HCC mouse model [48]. Recently, a comprehensive study in non-small cell lung cancer demonstrated that reduction in STAT3 in the tumor microenvironment using an antisense oligonucleotide reversed immunotherapy resistance in preclinical STK11 knockout mouse models [49]. Supporting these data, inhibition of the AURKA/STAT3 signaling pathway promoted effective T-cell infiltration into the tumor microenvironment and improved anti-PD-1 efficacy [50]. Therefore, a combination of STAT3-inhibition and immune therapy may be beneficial to HCC patients [31,45]. However, clinical studies are not available thus far and the role of STAT1 and STAT3 is not yet fully understood [51]. Thus, it will be crucial to evaluate different approaches to inhibit STAT3 signaling in the tumor cells and in the tumor microenvironment alone or in combination with immune therapy.

## 5. Conclusions

In conclusion, we show that in the absence of STAT3, IL-6 induced prolonged STAT1 signaling and expression of STAT1 target genes, which suggests an interplay of STAT1 and STAT3 signaling in the presence of proinflammatory IL-6 in the tumor microenvironment. Furthermore, activation of STAT1 and STAT3 in the tumor cells strongly correlates with the activation of STAT1 and STAT3 in infiltrating immune cells and infiltration of CD4-, CD8-, and FOXP3-positive immune cells, indicating high immunological tolerance in a subset of HCC patients, as evident by PD-L1 expression. Therefore, approaches to inhibit STAT3 signaling alone or in combination with immune therapy may improve patient outcome.

## Data Availability

Additional datasets analyzed during the current study are available from the corresponding author upon reasonable request.

## Supplementary Materials

The following supporting information can be downloaded at: https://www.mdpi.com/article/10.3390/cancers14051154/s1, Figure S1: STAT1 depletion does not induce IL-6-like response in HCC cells; Figure S2: STAT3 depletion prolongs IL 6 induced STAT1 phosphorylation in HepG2; Figure S3: Pairwise correlation of nuclear STAT1 staining with infiltrating immune cells in human HCC tissues; Figure S4: Correlation of STAT1 and STAT3 expression with immune cell infiltration in CCA; Figure S5: Association between nuclear STAT3 expression in the tumor epithelium and immune cell infiltration in HCC tumor samples; Table S1: Clinicopathological characteristics of the HCC cohort (*N* = 124) and comparison of patients with STAT1 or STAT3 nuclear low or high staining; Table S2: Clinicopathological characteristics of the CCA cohort (*N* = 138) and comparison of patients with STAT1 or STAT3 nuclear low or high staining; Table S3: siRNAs used for gene silencing; Table S4: Primers used for qRT-PCR; Table S5: Antibodies used for Western blot (WB) and immunohistochemistry (IHC); Supplemental Whole Western Blot Figures.
