# Supplementary material for: STAT1 and STAT3 Exhibit a Crosstalk and Are Associated with Increased Inflammation in Hepatocellular Carcinoma
Source: Cancers (Basel). 2022 Feb 23;14(5):1154. doi: 10.3390/cancers14051154 (PMC8909292; doi:10.3390/cancers14051154)

Supplemental Information

STAT1 and STAT3 Exhibit a Crosstalk and Are Associated with Increased Inflammation in Hepatocellular Carcinoma

Supplemental material included:

Supplemental Figures......2

Figure S1: STAT1 depletion does not induce IL-6-like response in HCC cells......2

Figure S2: STAT3 depletion prolongs IL-6-induced STAT1 phosphorylation in HepG2......3

Figure S3: Pairwise correlation of nuclear STAT1 staining with infiltrating immune cells in human HCC tissues......4

Figure S4: Correlation of STAT1 and STAT3 expression with immune cell infiltration in CCA..5

Figure S5. Association between nuclear STAT3 expression in the tumor epithelium and immune cell infiltration in HCC tumor samples......6

Supplemental Tables......7

Table S1: Clinicopathological characteristics of the HCC cohort (N=124) and comparison of patients with STAT1 or STAT3 nuclear low or high staining[#]......7

Table S2: Clinicopathological characteristics of the CCA cohort (N=138) and comparison of patients with STAT1 or STAT3 nuclear low or high staining[#]......8

Table S3: siRNAs used for gene silencing......9

Table S4: Primers used for qRT-PCR......10

Table S5: Antibodies used for Western blot (WB) and immunohistochemistry (IHC)......11

Supplemental Whole Western Blot Figures......12

Supplemental Figures

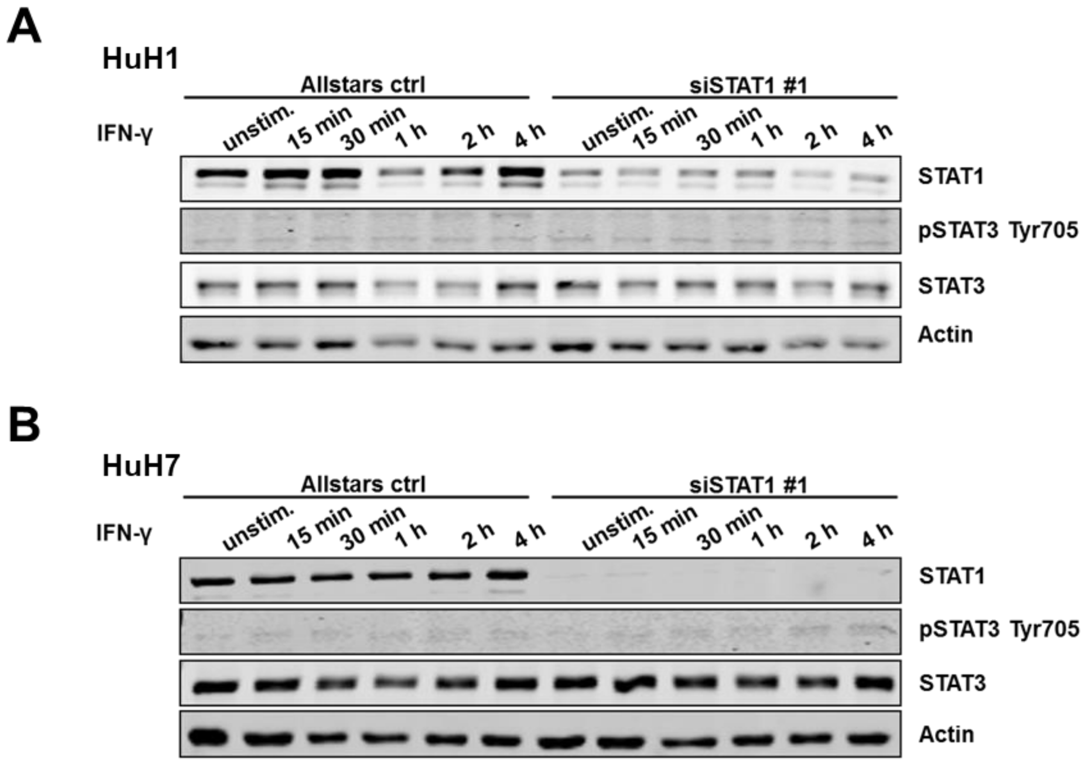

Figure S1: STAT1 depletion does not induce IL-6-like response in HCC cells. (A, B) Western blots detecting STAT1, STAT3 and pSTAT3 Tyr705 in Allstars control or STAT1-depleted **(A)** HuH1 and **(B)** HuH7 cells upon incubation with IFN-γ (500 U/ml) for indicated time points. Actin served as loading control.

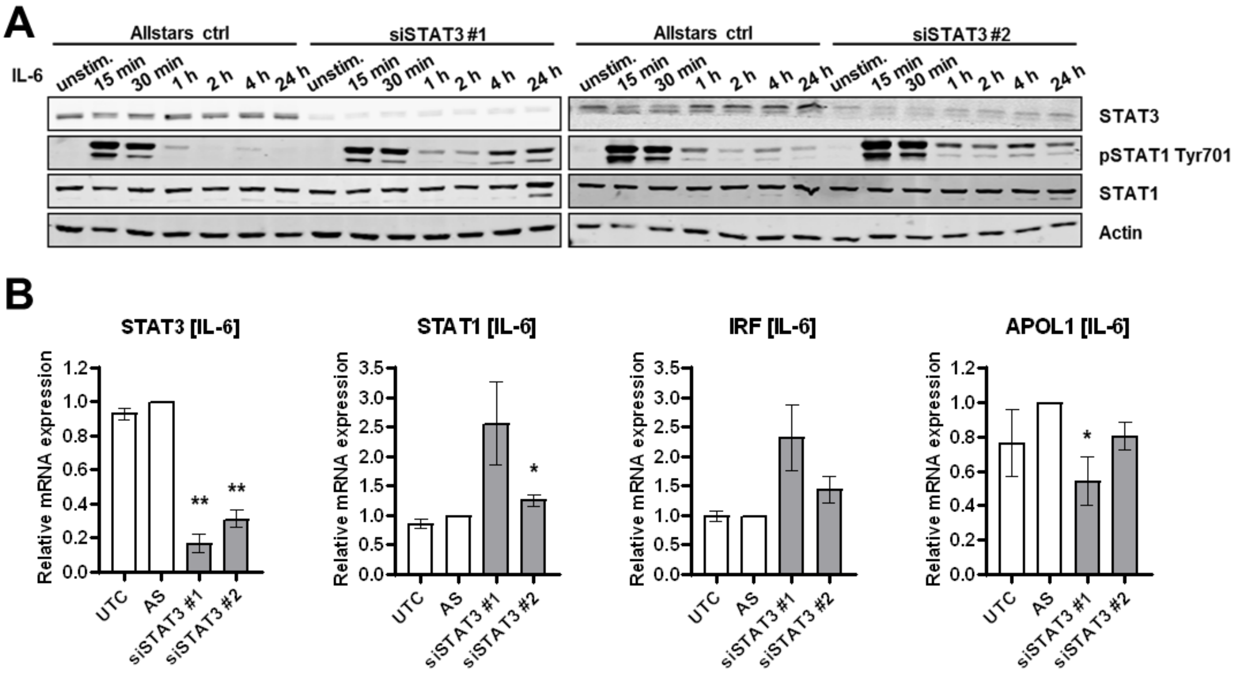

Figure S2: STAT3 depletion prolongs IL-6-induced STAT1 phosphorylation in HepG2. (A) Western blots of STAT3, pSTAT1 Tyr701 and STAT1 protein are shown. STAT3 was depleted using two different siRNAs, STAT3#1 and STAT3#2 in HepG2 cells. Cells were incubated with IL-6 (20 ng/ml) for indicated time points. Actin served as loading control. **(B)** Relative mRNA expression levels of STAT1 target genes *IRF1* and *APOL1* measured by semi-quantitative RT-PCR in untransfected control (UTC) HepG2 cells, cells transfected with Allstars (AS) control siRNAs, siSTAT3#1 or STAT3#2, as indicated, and incubated with IL-6 (20 ng/ml) for 24 h. N=3. * p<0.05, ** p<0.01.

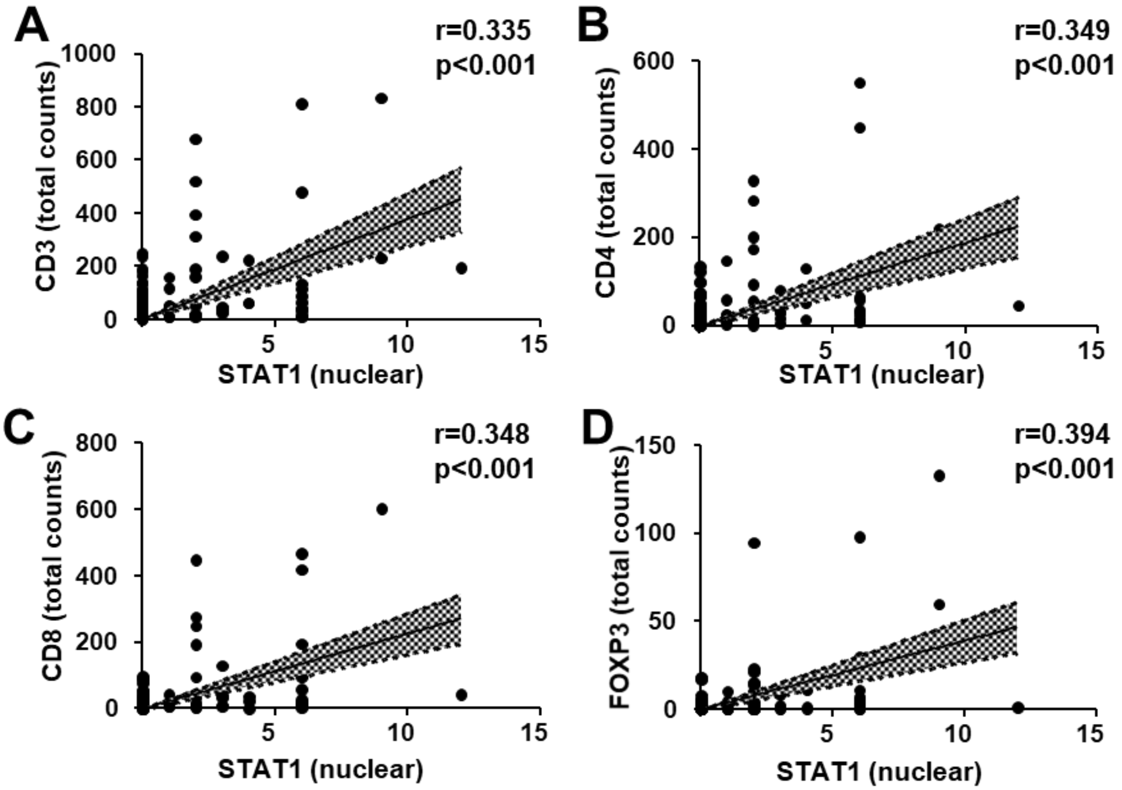

Figure S3: Pairwise correlation of nuclear STAT1 staining with infiltrating immune cells in human HCC tissues. (A-D) Spearman correlation plots of nuclear STAT1 staining, depicted as semi-quantitative immunoreactive score (IRS; expression values from 0 (no expression) to 12 (strong expression), with total counts of **(A)** CD3-postive, **(B)** CD4-positive, **(C)** CD8-positive or **(D)** FOXP3-positive tumor infiltrating immune cells in human HCC samples (N=109). Spearman correlation coefficient r and p-value are depicted for each correlation.

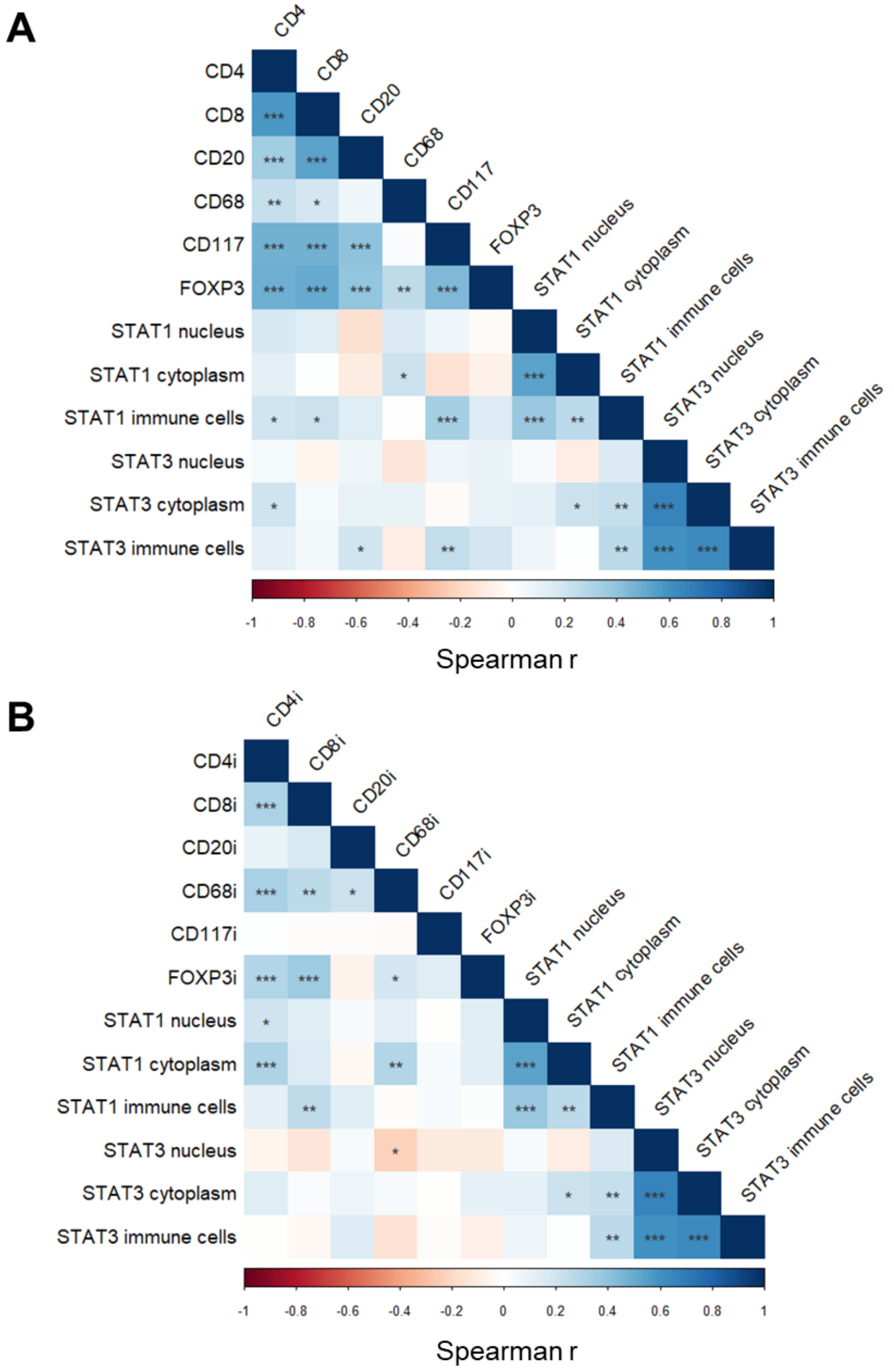

Figure S4: Correlation of STAT1 and STAT3 expression with immune cell infiltration in CCA. (A) Correlation matrix of total counts of immune cell infiltrates and immunohistochemical STAT1 or STAT3 expression in CCA tumor tissue samples. **(B)** Correlation matrix of intraepithelial counts of immune cell infiltrates and immunohistochemical STAT1 or STAT3 expression in CCA tumor tissue samples. Red color indicates negative correlation, whereas blue color indicates positive correlation. Darker coloration indicates a higher Spearman correlation coefficient and asterisk denote the level of significance. Spearman p-value: * $p<0.05$; ** $p<0.01$; *** $p<0.001$.

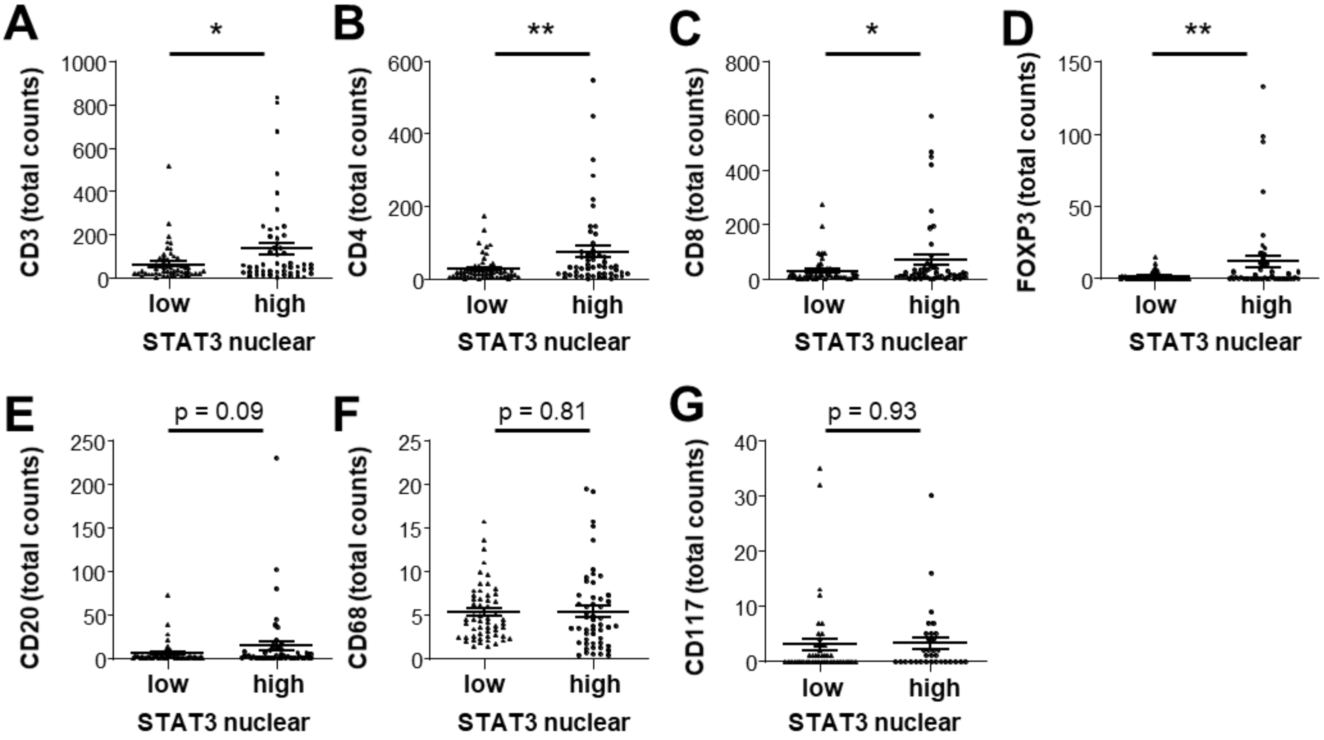

Figure S5. Association between nuclear STAT3 expression in the tumor epithelium and immune cell infiltration in HCC tumor samples. (A) CD3-, **(B)** CD4-, **(C)** CD8-, **(D)** FOXP3-, **(E)** CD20-, **(F)** CD68- and **(G)** CD117-positive immune cell infiltrate in HCC tumor tissue samples stratified by median score of nuclear STAT3 expression in the tumor epithelium into low (N=57) and high (N=52) groups. * $p<0.05$; ** $p<0.01$.

Supplemental Tables

Table S1: Clinicopathological characteristics of the HCC cohort (N=124) and comparison of patients with STAT1 or STAT3 nuclear low or high staining[#].

Characteristic	All cases	STAT1 low	STAT1 high	STAT1 NA	p-value*	STAT3 low	STAT3 high	STAT3 NA	p-value*
Age (years)									
=< 60	56	31	22	3	0.702	26	23	7	1.000
> 60	68	40	23	5		31	29	8	
Sex									
Male	105	64	34	7	0.063	53	41	11	0.050
Female	19	7	11	1		4	11	4	
Etiology									
Viral (HBV/HCV)	52	27	23	2	0.067	26	22	4	0.757
Alcoholic	29	23	6	-		15	12	2	
Other	43	21	16	6		16	18	9	
Liver disease									
Cirrhosis	68	40	28	-	0.815	36	29	3	0.243
Fibrosis	28	17	9	2		9	15	4	
None	28	14	8	6		12	8	8	
Nodularity									
uninodular	75	38	29	8	0.335	28	33	14	0.176
multinodular	49	33	16	-		29	19	1	
Vascular invasion									
V0	35	23	11	1	0.367	25	25	2	1.000
V1	58	29	23	6		25	24	9	
Grading									
G1	11	6	5	-	0.072	4	6	1	0.633
G2	82	51	23	8		39	33	10	
G3	26	13	13	-		11	12	3	
G4	5	1	4	-		3	1	1	

[#] Median cutoff was applied to compare low versus high STAT1 or STAT3 nuclear staining, respectively.

* Fisher's exact or chi-square test, as appropriate. Missing data were omitted.

Table S2: Clinicopathological characteristics of the CCA cohort (N=138) and comparison of patients with STAT1 or STAT3 nuclear low or high staining[#].

Characteristic	All cases	STAT1 low	STAT1 high	STAT1 NA	p-value*	STAT3 low	STAT3 high	STAT3 NA	p-value*
Age									
<60	52	31	12	9	1.000	24	20	8	0.849
>60	86	53	21	12		38	36	12	
Sex									
Male	89	54	22	13	1.000	30	46	13	**0.0002**
Female	49	30	11	8		32	10	7	
Subtype									
iCCA	61	37	16	8	0.058	38	15	8	**0.001**
pCCA	45	23	14	8		14	21	10	
dCCA	32	24	3	5		10	19	3	
Histology									
Ductal	107	67	28	12	0.856	45	51	11	0.433
Papillary	7	6	1	-		4	3	-	
Intestinal	2	1	1	-		2	-	-	
Mucinous	2	1	-	1		-	1	1	
Mixed	5	3	1	1		4	1	-	
Grading									
G1	6	2	3	1	0.075	3	2	1	0.804
G2	92	63	19	10		44	41	7	
G3	28	15	10	3		11	13	4	
pT									
T1	19	13	4	2	0.200	11	6	2	0.305
T2	74	45	21	8		34	32	8	
T3	26	20	4	2		9	15	2	
T4	7	2	3	2		4	2	1	
pN									
N0	48	30	15	3	0.809	18	27	3	0.819
N1	42	24	10	8		15	19	8	
pNx	48	30	8	10		29	9	10	
M									
M0	124	78	32	14	0.673	57	54	13	0.212
M1	11	5	1	5		5	1	5	
L									
L0	76	50	19	7	0.833	40	30	6	0.131
L1	54	31	13	10		19	26	9	
V									
V0	100	66	23	11	0.330	48	43	9	1.000
V1	34	17	10	7		14	13	7	

[#] Median cutoff was applied to compare low versus high STAT1 or STAT3 nuclear staining, respectively.

* Fisher's exact or chi-square test, as appropriate. Missing data were omitted.

Table S3: siRNAs used for gene silencing

Target	siRNA Name	Sequence 5‘-3‘	GeneGlobe ID	Supplier
None	Allstars	Negative control	NA	Qiagen
STAT1	siSTAT1 #1	CAGAAAGAGCTTGACAGTAAA	SI02662324	Qiagen
STAT1	siSTAT1 #2	CCAGATGTCTATGATCATTTA	SI02662884	Qiagen
STAT3	siSTAT3 #1	CAGCCTCTCTGCAGAATTCAA	SI02662338	Qiagen
STAT3	siSTAT3 #2	CAGGCTGGTAATTTATATAAT	SI02662898	Qiagen

NA: not applicable

Table S4: Primers used for qRT-PCR

Gene	Accession	Sequence 5’-3’	Amplicon
SRSF4	NM_005626.4	Forward: TGCAGCTGGCAAGACCTAAA<br>Reverse: TTTTTGCGTCCCTTGTGAGC	80 bp
STAT1	NM_001384880.1/<br>NM_001384881.1/<br>NM_001384882.1/<br>NM_001384883.1/<br>NM_001384884.1/<br>NM_001384885.1/<br>NM_001384886.1/<br>NM_001384887.1/<br>NM_001384888.1/<br>NM_001384889.1/<br>NM_001384890.1/<br>NM_001384891.1/<br>NM_007315.4 /<br>NM_139266.3	Forward: ACCTAACGTGCTGTGCGTAG<br>Reverse: GGTGAACCTGCTCCAGGAAT	129 bp
STAT3	NM_139276.2/<br>NM_003150.3/<br>NM_213662.1	Forward: ATCCTGGTGTCTCCACTGGT<br>Reverse: GTCTTCAGGTATGGGGCAGC	131 bp/ 128 bp
TTR	NM_000371.4	Forward: GCCGTGCATGTGTTCAGAAA<br>Reverse: AGCTCTCCAGACTCACTGGT	80 bp
SPINK1	NM_001354966.2/<br>NM_001379610.1/<br>NM_003122.5	Forward: CCTTGGCCCTGTTGAGTCTA<br>Reverse: AGTCCCACAGACAGGGTCATA	116 bp
IRF1	NM_002198.2/<br>NM_001354925.1<br>NM_001354924.1	Forward: ACCCTGGCTAGAGATGCAGA<br>Reverse: TGCTTTGTATCGGCCTGTGT	175 bp
APOL1	NM_001136540.2/<br>NM_003661.4/<br>NM_145343.2	Forward: ACAGCTGGATCTTGCTCAGTC<br>Reverse: CTCCCACACCAAGGAAAAGTG	135 bp

Table S5: Antibodies used for Western blot (WB) and immunohistochemistry (IHC)

Antigen (clone)	Dilution	Host species	Product/Company
**β-Actin**	WB: 1:10,000*	Mouse	691001/MP Biomedicals
**β-Tubulin**	WB: 1:500	Mouse	556321/BD Biosciences
**CD3 (SP7)**	IHC: ready-to-use	Rabbit	RBG026/Zytomed
**CD4 (4B12)**	IHC: 1:20	Mouse	NCL-L-CD4-368/Novocastra
**CD8 (C8/144B)**	IHC: 1:150	Mouse	M7103/DAKO
**CD20 (L20)**	IHC: 1:150	Mouse	M0755/DAKO
**CD68 (KP1)**	IHC: 1:8,000	Mouse	M0814/DAKO
**CD117**	IHC: 1:50	Rabbit	A4501/DAKO
**Foxp3 (236A/E7)**	IHC: 1:100	Mouse	ab20034/Abcam
**IRDye 680LT Donkey anti-mouse IgG (H + L)**	WB: 1:20,000	Donkey	926-68022/LI-COR Biosciences
**IRDye 680LT Donkey anti-rabbit IgG (H + L)**	WB: 1:20,000	Donkey	925-6802/LI-COR Biosciences
**IRDye 800CW Donkey anti-mouse IgG (H + L)**	WB: 1:20,000	Donkey	926-32212/LI-COR Biosciences
**IRDye 800CW Donkey anti-rabbit IgG (H + L)**	WB: 1:20,000	Donkey	926-32213/LI-COR Biosciences
**Stat1 (polyclonal)**	WB: 1:1,000	Rabbit	9172/Cell Signaling
**Stat1 (C-136)**	IHC: 1:200	Mouse	sc-464/Santa Cruz
**p-Stat1 (Tyr701) (D4A7)**	WB: 1:1,000	Rabbit	7649/Cell Signaling
**Stat3 (124H6)**	WB: 1:1,000	Mouse	9139/Cell Signaling
**Stat3 (79D7)**	IHC: 1:400	Rabbit	4904/Cell Signaling
**p-Stat3 (Tyr705) (D3A7) XP®**	WB: 1:1,000	Rabbit	9145/Cell Signaling

Supplemental Whole Western Blot Figures

To Figure 1B

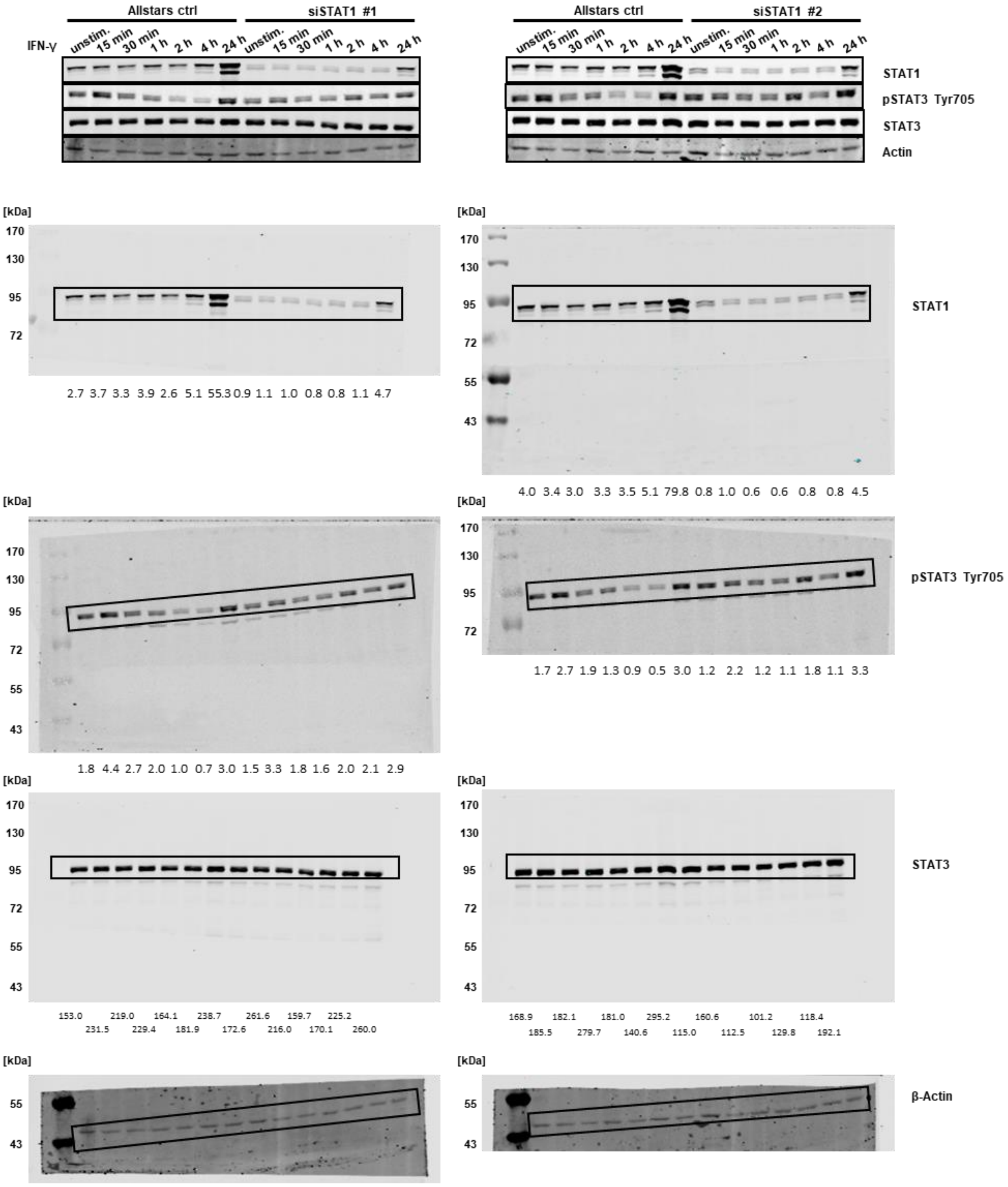

To Figure 2A

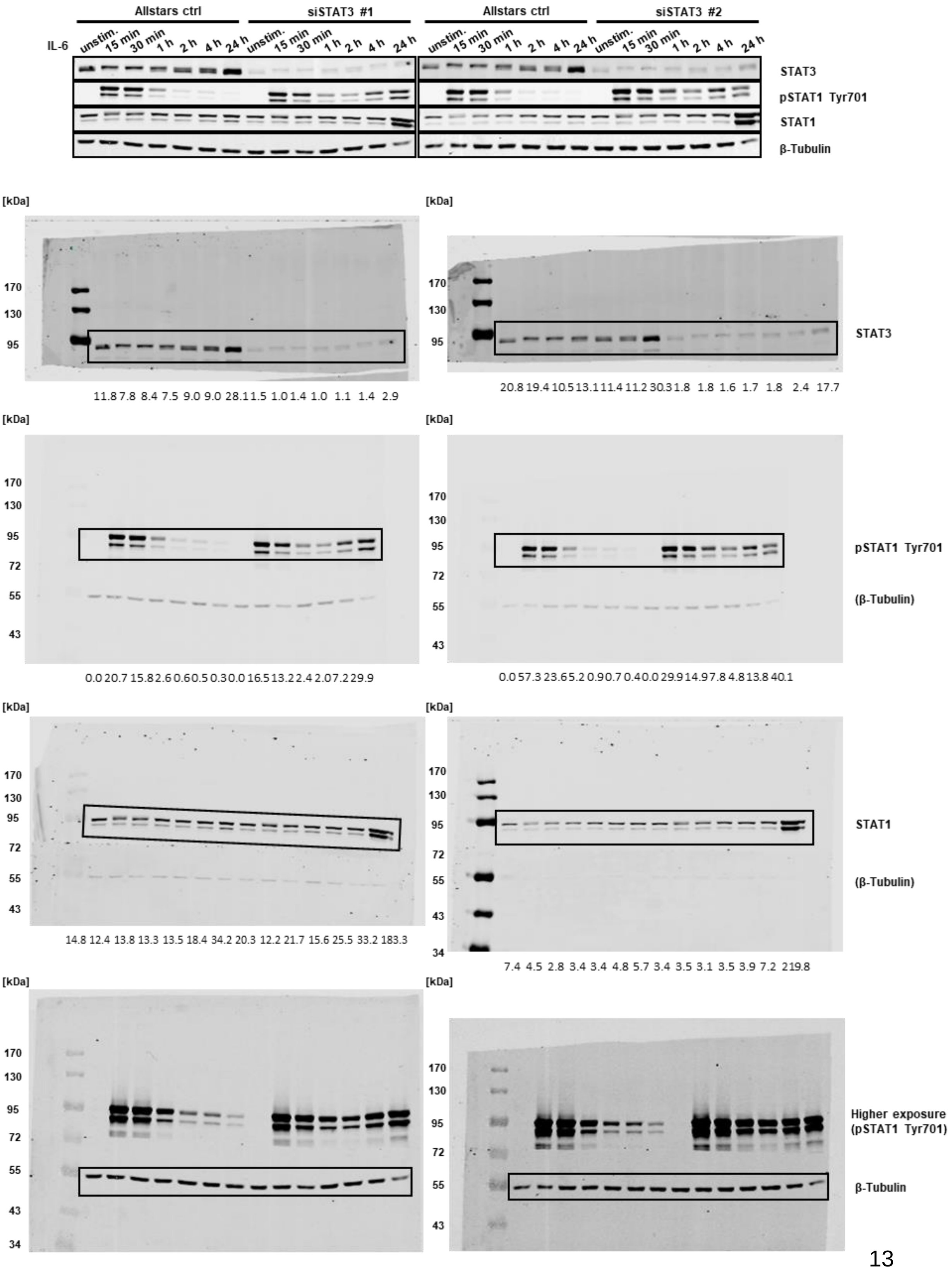

Allstars ctrl
siSTAT3 #1
Allstars ctrl
siSTAT3 #2
IL-6
unstim. 15 min 30 min 1 h 2 h 4 h 24 h unstim. 15 min 30 min 1 h 2 h 4 h 24 h unstim. 15 min 30 min 1 h 2 h 4 h 24 h unstim. 15 min 30 min 1 h 2 h 4 h 24 h
STAT3
pSTAT1 Tyr701
STAT1
β-Tubulin
[kDa]
170
130
95
11.8 7.8 8.4 7.5 9.0 9.0 28.1 1.5 1.0 1.4 1.0 1.1 1.4 2.9
20.8 19.4 10.5 13.1 11.4 11.2 30.3 1.8 1.8 1.6 1.7 1.8 2.4 17.7
STAT3
[kDa]
170
130
95
72
55
43
0.0 20.7 15.8 2.6 0.6 0.5 0.3 0.0 16.5 13.2 2.4 2.0 7.2 29.9
0.0 57.3 23.6 5.2 0.9 0.7 0.4 0.0 29.9 14.9 7.8 4.8 13.8 40.1
pSTAT1 Tyr701
(β-Tubulin)
[kDa]
170
130
95
72
55
43
34
14.8 12.4 13.8 13.3 13.5 18.4 34.2 20.3 12.2 21.7 15.6 25.5 33.2 183.3
7.4 4.5 2.8 3.4 3.4 4.8 5.7 3.4 3.5 3.1 3.5 3.9 7.2 219.8
STAT1
(β-Tubulin)
[kDa]
170
130
95
72
55
43
34
Higher exposure (pSTAT1 Tyr701)
β-Tubulin

To Figure 2B

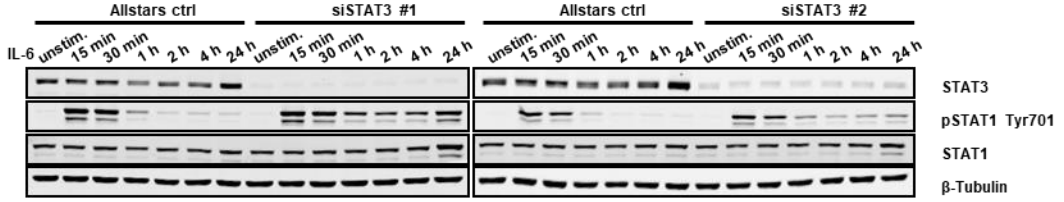
Allstars ctrl
siSTAT3 #1
Allstars ctrl
siSTAT3 #2
IL-6
unstim.
15 min
30 min
1 h
2 h
4 h
24 h
STAT3
pSTAT1 Tyr701
STAT1
β-Tubulin

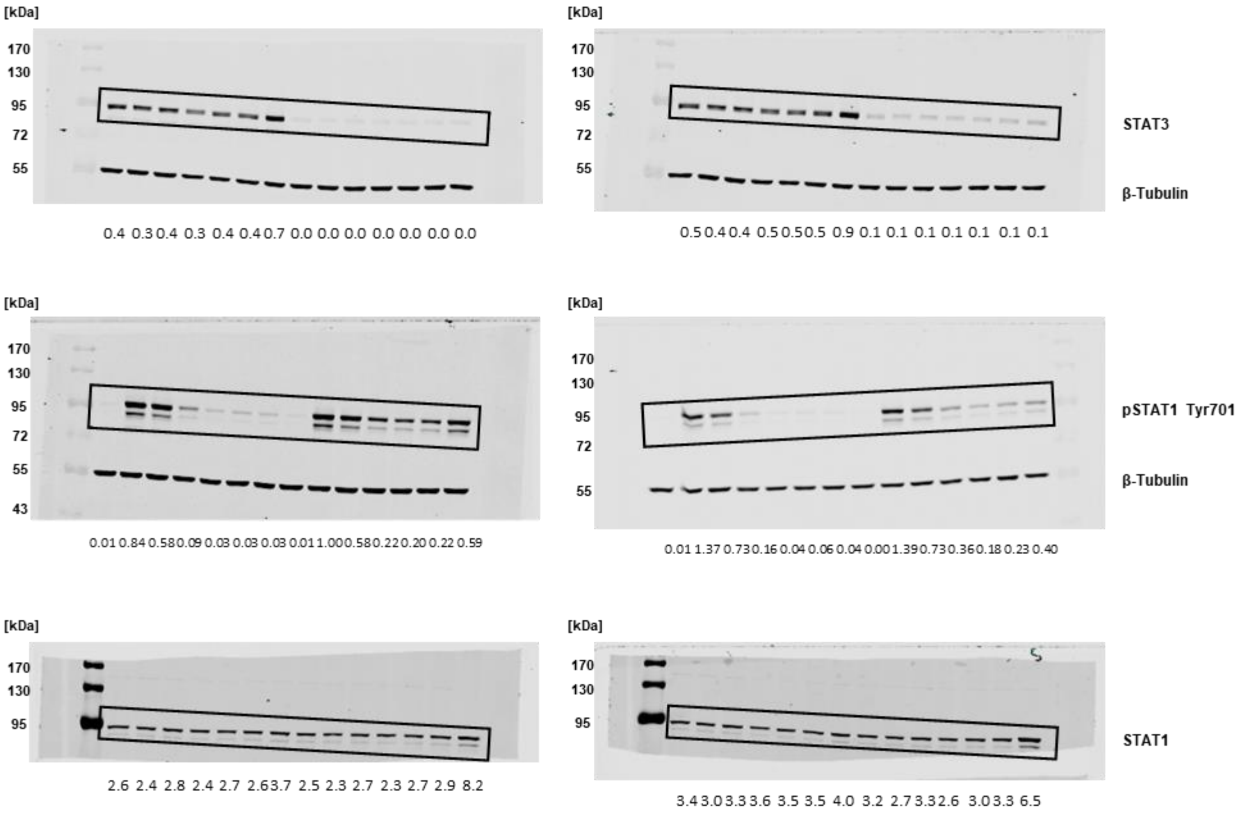
[kDa]
170
130
95
72
55
43
STAT3
β-Tubulin
pSTAT1 Tyr701
STAT1
0.4 0.3 0.4 0.3 0.4 0.4 0.7 0.0 0.0 0.0 0.0 0.0 0.0 0.0
0.5 0.4 0.4 0.5 0.5 0.5 0.9 0.1 0.1 0.1 0.1 0.1 0.1 0.1
0.01 0.84 0.58 0.09 0.03 0.03 0.03 0.01 1.00 0.58 0.22 0.20 0.22 0.59
0.01 1.37 0.73 0.16 0.04 0.06 0.04 0.00 1.39 0.73 0.36 0.18 0.23 0.40
2.6 2.4 2.8 2.4 2.7 2.6 3.7 2.5 2.3 2.7 2.3 2.7 2.9 8.2
3.4 3.0 3.3 3.6 3.5 3.5 4.0 3.2 2.7 3.3 2.6 3.0 3.3 6.5

To Figure S1A+B

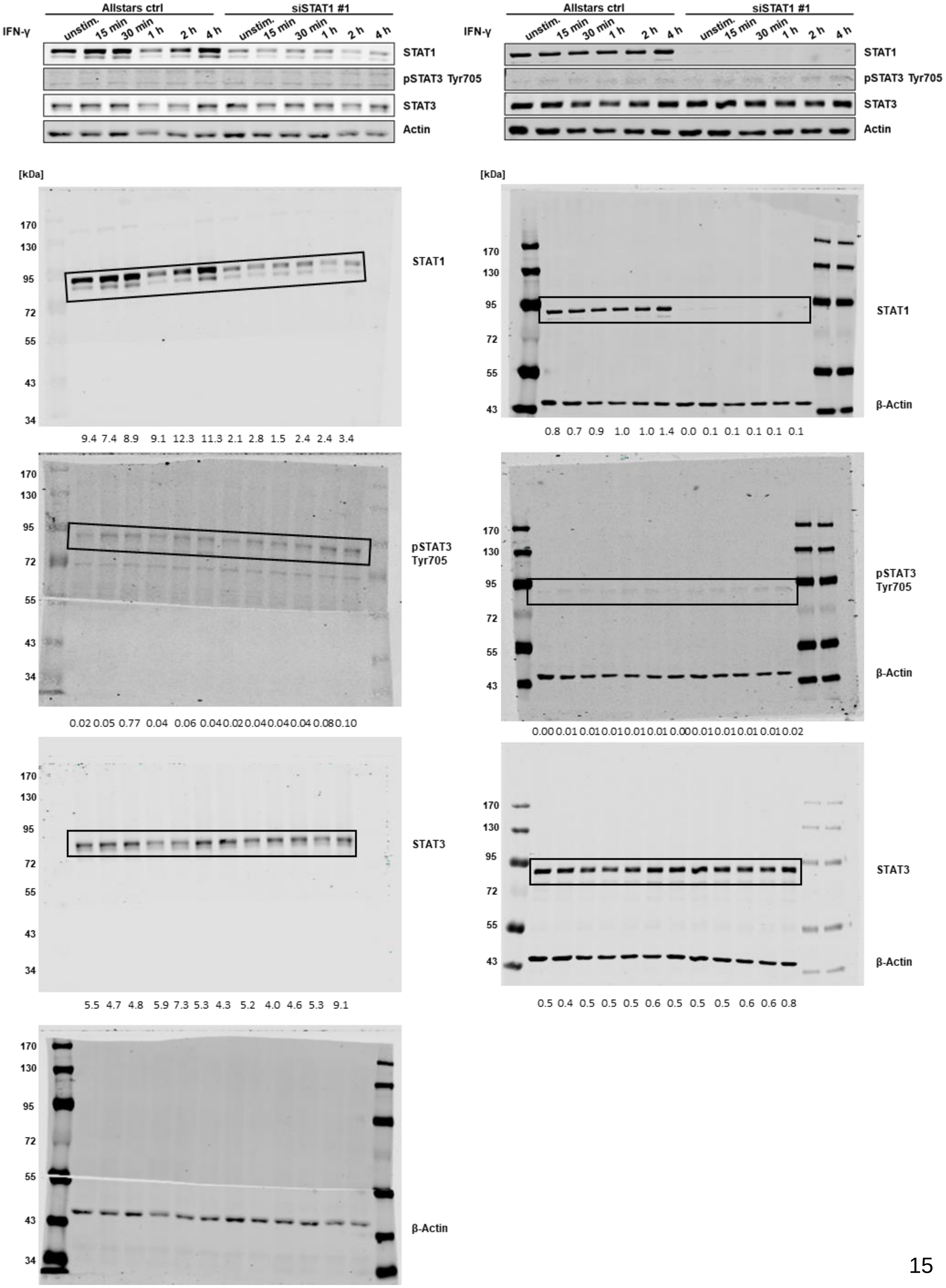

To Figure S2A

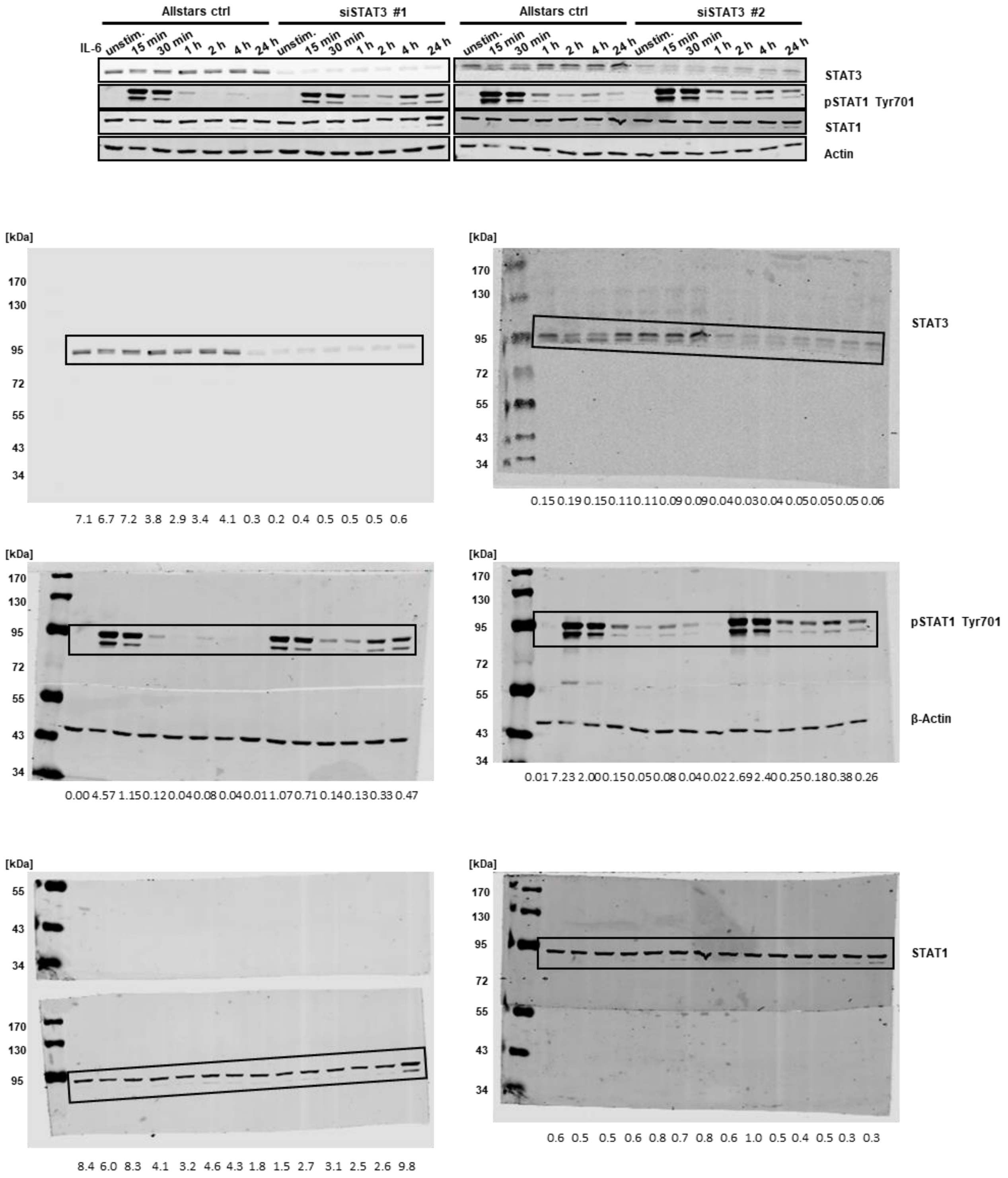

Supplement: Supplementary file 1 [file cancers-14-01154-s001.zip › cancers-1592992-supplementary.pdf]
